# Association of antenatal care and place of delivery with newborn care practices: evidence from a cross-sectional survey in rural Uttar Pradesh, India

**DOI:** 10.1186/s41043-017-0107-z

**Published:** 2017-06-21

**Authors:** Niveditha Devasenapathy, Sutapa B. Neogi, Srinivasan Soundararajan, Danish Ahmad, Avishek Hazra, Jaleel Ahmad, Neelakshi Mann, Dileep Mavalankar

**Affiliations:** 1 Indian Institute of Public Health-Delhi, Plot No. 47, Sector 44, Institutional Area, Gurgaon, 122002 India; 2 Uttar Pradesh Community Mobilization Project, Public Health Foundation of India, Gurgaon, 122002 India; 3 Population Council, Zone 5A, Ground Flr, India Habitat Centre,, Lodhi Road, New Delhi, 110003 Delhi India; 4 Rajiv Gandhi Mahila Vikas Pariyojana (RGMVP), Kanpur Road, Rana nagar, Raebareli, (UP)-229001 India; 5 Indian Institute of Public Health Gandhinagar, Opp. Air Force Headquarters, Palej Road, Sector 30, Gandhinagar, Gujarat India

**Keywords:** Newborn care, Antenatal care, Delivery, Community health workers

## Abstract

**Background:**

Appropriate immediate newborn care is vital for neonatal survival. Antenatal period is a crucial time to impart knowledge and awareness to mothers regarding newborn care, either during facility visits or during home visits by community health workers (CHWs) especially in the rural context. In this paper, we report newborn care practices in rural Uttar Pradesh (UP) and have explored association between newborn care practices with antenatal care, contact with community health workers during pregnancy and place of childbirth.

**Methods:**

We use cross-sectional baseline data (which is part of a larger intervention project) collected from 129 gram panchayats (GPs) from 15 administrative blocks spread over five districts of UP in 2013. From currently married women (*n* = 2208) of 15–49 years, who delivered 15 months prior to the survey, we collected information on women’s demographic and socio-economic characteristics, knowledge and practice of reproductive, maternal, newborn, child health and nutrition behaviours. Association of newborn practices with antenatal care, contacts by community health worker during pregnancy and place of childbirth were tested using random intercept logistic regression, adjusting for socio-economic and demographic factors and accounting for clustering at the GP and block levels.

**Results:**

Eighty-three percent of 2208 mothers received ANC, but only half of the respondents received a minimum of three ANC visits. More than two thirds of respondents delivered at a health facility. Practice of newborn care was poor: merely one fourth of women practised clean cord care, one third of women followed good breastfeeding practices (initiation with an hour of birth, fed colostrum and did not give pre-lacteal feeds) and one third provided adequate thermal care (kept baby warm and delayed bathing). Only 5% followed all above practices with evidence of clustering of newborn care practices at the block and GP levels. While facility-based childbirth was strongly associated with appropriate newborn care practices, ANC visits and contacts with CHWs was not associated with all newborn care practices.

**Conclusion:**

The quality of ANC care provided needs to be improved to have an impact on newborn care practices. Our finding emphasizes the importance of facility-based birthing. There is a need for training CHWs to strengthen their counselling skills on newborn care. Variation of newborn care practices between communities should be taken into consideration while implementing any intervention to optimize benefits.

## Key messages

- Only 5% of mothers practised all three newborn care practices, namely timely initiation of breast feeding, thermal care and clean cord care.
- Facility-based deliveries are associated with better newborn care practices as compared to home delivery.
- There is a need to emphasize on the quality of counselling during antenatal care (ANC) visits and home visits by community health workers to increase adoption of preventive newborn care practices by newly delivered mothers.
- Significant heterogeneity in newborn practices across blocks and smaller units (gram panchayats) exists. Need to identify cluster-level factors for better implementation of programmes and interventions.

## Background

Major causes for neonatal deaths are due to preterm births and intrapartum complication [1]. While adequate antenatal care and delivery at health facility have shown to reduce stillbirths and is vital for safe mother–newborn dyad; clean cord care, thermal care and appropriate timely initiation of breast-feeding practices are important contributing factors for reduction in newborn morbidity and mortality [2, 3]. As per WHO guidelines, initiation of breast feeding soon after birth, prevention of hypothermia and infections and clean cord care are recommended for all newborns. Use of chlorhexidine may be considered only to replace application of harmful substances, such as cow dung to the cord stump [4]. Antenatal period is the ideal time to impart knowledge regarding appropriate immediate newborn care practices when the women visit hospitals and during home visits made by community health workers. However, studies have shown that in the context of developing countries, antenatal care interventions or completing adequate number of ANC visits may not be associated with lower risk of neonatal death [5, 6]. This could be due to a gap between coverage and quality of ANC. Delivery at hospitals and a good intrapartum care have shown to have impact on newborn care practices. A nationally representative study from India has shown that facility-based delivery followed by adequate postnatal check-ups substantially reduced neonatal deaths as compared to mothers that had just facility delivery without any postnatal check-up [7] emphasizing the importance of postnatal care in the early newborn period. Another study by Khan et al. [8] in Uttar Pradesh showed that the financial incentives for delivering at a health facility increased client–provider contact, uptake of minimum of three ANC visits and facility-based delivery. These practices together provide windows of opportunity for providing counselling and advice which, in turn, trigger the adoption of a cluster of healthy behaviours that have a direct bearing on maternal and child health.

There is a decreasing trend in neonatal mortality across Indian states. As per Sample Registration System (SRS), the neonatal mortality rate (NMR) (2012) for Uttar Pradesh is 39 per 1000 live births which contributes to 27% of India’s total NMR burden [9, 10]. Among the rural population, 34% receive at least three ANC visits, 60% of women give birth at a health facility and only one in five mothers receive any postnatal check-up [11]. The percentage of rural pregnant women that received the recommended four antenatal check-ups is 21.4% as reported by the recent National Family Health Survey 2015-16 (NFHS-4) [12]. Furthermore, the coverage of these indicators vary widely across the 72 districts of Uttar Pradesh. The poor and disadvantaged section of society are the most likely to have limited access to optimal health care leading to poorer maternal and child health outcomes [13]. In rural Uttar Pradesh (UP), Rajiv Gandhi Mahila Vikas Pariyojana (RGMVP) has established a large base of volunteer-led self-help groups (SHGs), mostly constituted by poor and lower caste women, who work for poverty alleviation. To address the high burden of NMR, a health programme named “Uttar Pradesh Community Mobilization (UPCM) project” was initiated in 2011, funded by the Bill & Melinda Gates Foundation. This was led by the Public Health Foundation of India (www.phfi.org), with a goal to reduce infant mortality by improving maternal and child health behaviour, through scaling up health intervention packages via SHG using behaviour change communication methods. The learning phase of the larger project was established across eight districts (10 blocks and 100 gram panchayats) in 2013.

In this paper, we focus on three newborn care practices: clean cord care, timely initiation of breastfeeding practices and thermal care. We (i) describe newborn care practices as reported by mother’s recall and quantify the correlation of such practices within communities at block and gram panchayat (GP) level and (ii) explore the association between newborn care practices with ANC during pregnancy, number of contacts with community health workers during ANC period and place of childbirth.

## Methods

This study uses baseline data of the learning phase of the abovementioned UPCM project. This survey was conducted by Population Council, Delhi, during June to August, 2013, in 15 blocks from five districts chosen purposively based on geographical diversity. These districts had the presence of self-help groups that concentrated on health-related activities implemented by Rajiv Gandhi Mahila Vikas Pariyojana (RGMVP), a rights-based organization that works for poverty reduction, women’s empowerment and rural development in Uttar Pradesh (www.rgmvp.org/).

### Sampling and sample size

GPs were smaller administrative units within these blocks. A house listing of all the SHG members was done in all the listed GPs to identify eligible women from SHG households. To identify eligible women from non-SHG households in the intervention area, a listing of non-SHG households was done in the neighbourhood of SHG households to ensure similar socio-economic characteristics of women in both the groups. The required number of non-SHG households (approximately half of the sampled SHG households) was randomly selected from each villag*e.* The eligibility criteria for inclusion of respondents in the sampling frame were (a) women of age 15 to 49 years, (b) currently married and (c) had delivered a baby in the 15 months prior to the survey. Since only one woman represented each household, we consider women nested within GPs and GPs nested within blocks. Sample size calculations were meant for the objective to measure the changes in maternal neonatal and child health indicators over time in intervention area as compared to control area [14], and hence, there are no pre hoc sample size calculations for this exploratory analysis. For this paper, we have used the overall data from all blocks for presenting the results of study objectives.

### Data collection

After obtaining verbal informed consent from the mother, two sets of questionnaires, a household schedule and a woman schedule, were administered in Hindi by trained data collectors using a computer-assisted personal interview (CAPI) package designed using CSPro programme [15]. Information on the family members, socio-demography, household-level wealth and membership with any SHG were obtained. The women’s questionnaire collected information on antenatal, childbirth and postnatal care practices. There were no refusals, but a 25% non-response rate was documented mostly due to the respondents being away from the village on the day of the survey or the houses of the selected respondents were found locked on repeated visits. The percentage of non-availability of respondents at the time of survey was similar across the blocks. Finally, data were collected from 2208 women of which 1709 women belonged to SHG households and 499 belonged to non-SHG households.

### Statistical analysis

All analyses were performed using Stata14 (Statacorp, USA). Description of socio-demographic profile of household and mothers are presented using descriptive statistics for overall sample and categorized by SHG membership of the household. Data from mothers and their households was considered as the smallest unit, who were nested within a GP which in turn were nested within blocks. Proportion following key newborn care practices are presented along with intracluster correlation coefficient (ICC) at block level and GP level.

**Fig. 1:**
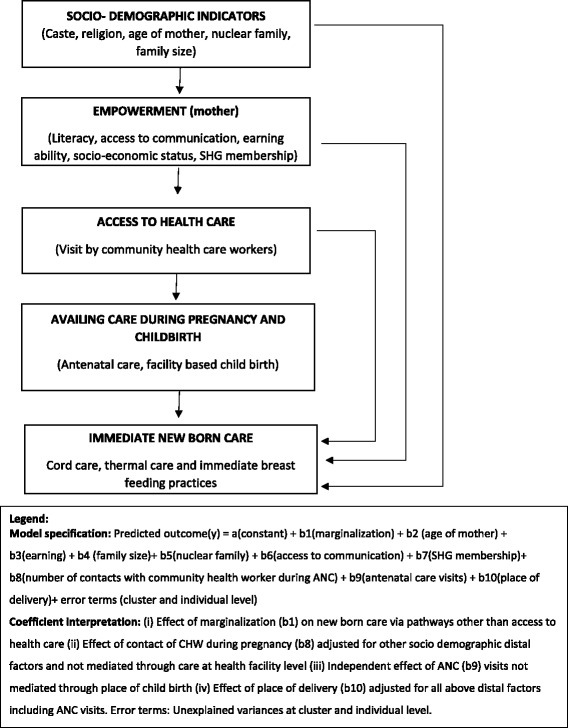
Conceptual framework: determinants for newborn care by rural women

We computed the socio-economic score of households using principal component analysis with data of household facilities and assets at the time of interview. We categorized the score into quintiles with the first quintile being the least poor and the last quintile the poorest of the poor. We defined levels of marginalization using three indicators viz. ability to read or write, caste and socio-economic status (SES) of the household. Women belonging to scheduled caste or tribe (SC/ST) category, unable to read or write and belonging to the last two quintiles of SES were considered as the most marginalized. Least marginalized were those who had none or only one of the marginalization factors described above. The definition of newborn care practices and other variables used in the analysis is found in Table 1. To explore the determinants of good newborn care practices, we performed multilevel random intercept logistic regression and present odds ratio (OR) with 95% CI. We used multilevel random intercept logistic regression as opposed to standard logistic regression technique as the data from mothers were nested within GP and blocks leading to clustering of outcomes. This enables appropriate estimation of standard errors around the estimate. Further by the random intercept method, we allow each cluster to have its own intercept but assume the slope to be the same for all clusters. The variables to be included in the model were based on statistical significance on univariable analysis and our previous experience in this field. We also explored the interaction between marginalization and SHG membership on newborn care indicators. Figure 1 depicts the conceptual framework developed before performing the analysis.

**Table 1:** Definition of certain exposure and outcome variables

*Marginalization*: This consists of three components: social class, literacy and socio-economic status. Women belonging to the SC/ST class, with no ability to read and write and belonging to the last two quintiles of the socio-economic score, were defined as “most marginalized”. Women with only two of the above indicators are classified as “some marginalization”, and those with only one or none would fall under “least marginalized”.
*Access to communication*: Women who had access to any one of the following: TV, radio or newspaper, were considered to have access to means of modern communication.
*SHG membership*: The respondent or one of her household members is a member of SHG run by Rajiv Gandhi Mahila Vikas Pariyojana.
*Antenatal visits*: 0 = who did not receive any ANC during last pregnancy, 1 = 1 or 2 visits, 2 = 3 and more visits either at a health facility or ANC received on Village Health and Nutrition Day (VHND) by auxiliary nurse midwife (ANM).
*Home delivery*: All women who gave birth at home (planned and unplanned).
*Good thermal care*: Those who kept baby warm by any method and those who delayed first bath of newborn beyond 48 h
*Clean cord care*: Those who did not apply any foreign material on the cord.
*Good breast-feeding practices*: Those who initiated breast feeding within 1 h of birth, did not discard colostrum and did not give any pre-lacteal feeds.

## Results

The average number of GPs per block interviewed was 8.6 (SD 1.8) (range 4–10). The number of women by GP and block along with household and mother’s socio-demographic profile for the overall sample and by SHG membership is presented in Table 2. Most characteristics were similar across SHG and non-SHG households. The overall study population were predominantly Hindus, half of them belonging to SC/ST and a mean household size of 7.2. Only half of the mothers had ever attended school and only 15% of those who attended till 4th standard could read. Less than half had some access to communication, and one third of these women had their own cell phones.

**Table 2:** Household and target women characteristics

Household characteristics	Overall	Target women are SHG members or belong to a SHG household	Target women not SHG members or belong to SHG household
	*N* = 2208 GP = 129 Block = 15	*n* = 1709 GP = 129 Block = 15	*n* = 499 GP = 68 Block = 9
Number of participants per block, mean (SD) (range)/block	147 (68) (36–309)	146 (73) (36–309)	153 (21) (136–168)
Number participants per GP, mean (SD) (range)	17.1 (10.8) (1–67)	17 (10.9) (1–67)	16 (7.6) (10–29)
Hindu religion, *n* (%)	2046 (92.7)	1586 (92.3)	460 (92.2)
Scheduled caste/tribe, *n* (%)	1172 (53.1)	945 (55.3)	227 (45.5)
Drinking water source, *n* (%)			
Pump/tube well	2013 (91.2)	1565 (91.6)	448 (90.0)
Open source (well/lake, etc.)	148 (6.7)	111 (6.5)	37 (7.4)
Piped (house/yard)	47 (2.13)	33 (1.9)	14 (2.8)
Toilet, *n* (%)			
Flush	133 (6.02)	94 (5.5)	39 (7.8)
Pit	43 (2)	30 (1.8)	13 (2.6)
Open defecation	2032 (92.03)	1585 (92.7)	447 (89.6)
Cooking fuel type, *n* (%)			
Electricity/LPG/kerosene	59 (2.7)	40 (2.3)	19 (3.8)
Wood/dung/shrub	2149 (97.3)	1669 (97.7)	480 (96.2)
Source of lighting, *n* (%)			
Electricity	572 (25.9)	434 (25.4)	138 (27.7)
Non-electricity	1636 (74.1)	1275 (74.6)	361 (72.3)
Number of hours of power supply, mean (SD)	7.16 (2.8)	7.16 (2.8)	7.17 (2.8)
Dwelling type, *n* (%)			
Thatched (Kuccha)	822 (37.2)	653 (38.2)	169 (33.9)
Semi-concrete (semi-Pucca)	1227 (55.6)	946 (55.4)	281 (56.3)
Concrete (Pucca)	159 (7.2)	110 (6.4)	49 (9.8)
Household size, mean (SD)	7.2 (2.8)	7.4 (3.05)	6.8 (3.02)
Nuclear type of family, *n* (%)	992 (44.9)	739 (43.2)	253 (50.7)
Socio-economic position^a^, *n* (%)			
0 (least poor)	440 (20)	315 (18.4)	125 (25.1)
1	443 (20.1)	355 (20.8)	88 (17.6)
2 (middle)	441 (20)	333 (19.5)	108 (21.6)
3	400 (18.1)	327 (19.1)	73 (14.6)
4 (poorest)	484 (21.9)	379 (22.2)	105 (21.04)
Women characteristics	*N* = 2208	SHG households *n* = 1709	Non-SHG *n* = 499
Age in years, mean (SD)	25.6 (4.9)	25.6 (4.9)	25.4 (5)
Ever attended school, *n* (%)	1155 (52.3)	890 (52.1)	265 (53.1)
Those who attended till 4th, *n* (%)			
Cannot read or write	888 (76.1)	691 (76.2)	197 (75.8)
Can read	186 (15.9)	152 (16.8)	34 (13.1)
Can read and write	93 (8)	64 (7.1)	29 (11.2)
Age in years started cohabiting with husband, mean (SD)	17.4 (2.3)	17.4 (2.3)	17.5 (2.3)
Working (cash/kind), *n* (%)	457 (20.7)	376 (22)	81 (16.2)
Access to communication, *n* (%)			
Do not read news paper	1832 (83)	1430 (83.7)	402 (80.6)
Do not listen to radio	1754 (79.4)	1351 (79.1)	403 (80.8)
Do not watch TV	1384 (62.6)	1086 (63.6)	298 (59.7)
Access to any one of the above mode of communication	987 (44.7)	756 (44.2)	231 (46.3)
Cell phone access, *n* (%)			
Own phone	739 (33.5)	561 (32.8)	178 (35.5)
Husband phone	948 (42.9)	752 (44.0)	196 (39.3)
Other’s phone	309 (14)	248 (14.5)	61 (12.2)
Do not use	212 (9.6)	148 (8.7)	64 (12.8)
Marginalization^a^, *n* (%)			
Least	827 (37.45)	608 (35.6)	219 (439)
Some	901 (40..8)	713 (41.7)	188 (37.7)
Most	480 (21.7)	388 (22.7)	92 (18.4)
Number of live births, median (IQR)	3 (1, 4)	3 (1, 4)	2 (1, 4)
Number of living children, median (IQR)	2 (1, 3)	2 (1, 3)	2 (1, 3)
At least one still birth, *n* (%)	138 (6.25)	104 (6.1)	34 (6.8)

^a^Marginalization defined as a composite of caste (SC/ST = 1, others = 0), literacy (cannot read and write = 1, only read/both = 0), socio-economic status (0–4 least poor to poorest) (0/2 = 0, 3/4 = 1). Total score ranges from 0 to 3. 3 = most marginalized, 2 = some marginalization, 1 and 0 least marginalized

### Care during pregnancy

Most women (84%) had received at least one ANC visit. The mean number of ANC visits overall for the sample was 2.6 (SD 1.9). Fifty-one percent had three or more visits and 91% of the women reported at least one contact with any of the community health workers (CHWs: auxiliary nurse midwife (ANM) or Accredited Social Health Activist (ASHA) or Anganwadi worker (AWW)) during pregnancy. Thirty-two percent of the women gave birth at home. Among those who delivered at a health facility, around 45% stayed at least for 24 h in the facility post-childbirth. Eight percent of mothers neither received ANC nor went to a health facility for childbirth. Around three fourths of mothers reported to have not received postnatal check-up for them or their newborn. Table 3 describes the self-reported items by recall, of advice given to them regarding postnatal care in the health facility before discharge.

**Table 3:** Information on last live childbirth as reported by recall of mother

Details last pregnancy and childbirth	*N* = 2208
Received at least one ANC check-up last pregnancy, *n* (%)	1842 (83.4)
Number of ANC visits, mean (SD)	2.5 (1.9)
Women with at least four ANC, *n* (%)	462 (20.9)
Had contact with any of the community health workers (CHWs) during ANC, *n* (%)	2007 (90.9)
Number of contacts with CHWs during ANC, median (IQR)	5 (3, 7)
Place of childbirth (%)	
Home	701 (31.8)
Health centre	937 (42.4)
Hospital	570 (25.8)
Planned place of childbirth, *n* (%)	1682 (76.2)
Vital status of offspring at time of interview (alive), *n* (%)	2189 (99.1)
Male child, *n* (%)	1115 (50.5)
Normal delivery, *n* (%)	1349 (90.2)
Instrumental, *n* (%)	65 (4.4)
LSCS, *n* (%)	81 (5.4)
Duration of stay in days in the hospital/health centre, median	0.62 (0.12,2)
(IQR) stayed at least for 24 h (1492), *n* (%)	672 (45.0)
Received advice was given before discharge (1495), *n* (%)	
Initiation of breast feeding within 1 h	
Feeding colostrum	1176 (78.7)
Exclusive breast feeding	1213 (81.1)
Kangaroo mother care	1040 (69.6)
Bathing after 24 h	311 (20.8)
Information on danger signs	579 (38.7)
Mother	542 (36.3)
Newborn	579 (38.7)
Contraception due to breast feeding	72 (4.8)
Postnatal care, *n* (%)	
None after childbirth	1628 (73.7)
Within 42 days but after 7 days of childbirth	199 (9)
Within 7 days of childbirth	381 (17.3)
Number of visits made (*n* = 580), median (IQR)	1 (1, 2)
Median time in days of first PNC visit (*n* = 580), median (IQR)	5 (1, 12)
Home visit by community health worker after childbirth, *n* (%)	
Within 42 days	1078 (48.8)
After 42 days	222 (10.1)
Not visited	908 (41.1)
Number of contacts within 42 days of childbirth, median (IQR) (n = 1300)	2 (1, 4)

### Newborn care

Newborn care practices (cord care, timely initiation of breast feeding and thermal care) as reported by the mothers by recall are reported in Table 4. We found small yet significant amount of clustering of these practices due to unexplained individual- or cluster-level factors, ranging from 2 to 6% at the block level and 5 to 11% at the GP level. Only 5% of mothers reported to have followed all the mentioned newborn practices.

**Table 4:** Newborn care practices followed during last childbirth and their clustering at the block and gram panchayat levels

Newborn care practices	Home deliveries	Delivery at a health care facility	Overall	ICC (95% CI)^a^	ICC (95% CI)^a^ gram panchayat
	(*n* = 701)	(*n* = 1507)	(*n* = 2208)	Block (*n* = 15)	(*n* = 129)
Cord care, *n* (%)					
Sterile thread used for tying cord	582 (83.0)	–	–	0.138 (0.047, 0.339)	0.237 (0.116, 0.423)
No application of foreign material on cord	119 (17.0)	463 (30.7)	582 (26.46)	0.04 (0.013, 0.115)	0.11 (0.066, 0.18)
Breast feeding, *n* (%)					
Initiated breast feeding within 1 h of birth	241 (34.4)	831 (55.1)	1072 (48.6)	0.04 (0.016, 0.092)	0.05 (0.023, 0.11)
Time in hours to initiate breast feeding median (IQR)	2 (1, 24.3)	1 (0.5, 3)	1.08 (0.67, 5)		
Did not discard colostrum	506 (72.2)	1330 (88.3)	1836 (83.15)	0.025 (0.007, 0.082)	0.058 (0.026, 0.124)
Did not give pre-lacteal feeds	228 (32.5)	984 (65.3)	1212 (54.9)	0.042 (0.016, 0.11)	0.115 (0.073, 0.175)
Did all three	126 (18.0)	634 (42.1)	760 (34.4)	0.05 (0.019, 0.112)	0.076 (0.04, 0.139)
Thermal care, *n* (%)					
Kept baby warm	485 (69.2)	1008 (66.9)	1493 (67.6)	0.027 (0.006, 0.098)	0.102 (0.063, 0.161)
Delayed first bath of baby by 48 h	140 (20.0)	933 (62.0)	1073 (48.6)	0.067 (0.03, 0.144)	0.074 (0.034, 0.154)
Did both	105 (15.0)	654 (43.4)	759 (34.4)	0.045 (0.016, 0.116)	0.099 (0.057, 0.165)
All 6 good newborn care practices^b^ *n* (%)	7 (1.0)	109 (6.8)	109 (4.9)	–	

^a^Intracluster correlation coefficient (ICC) from random intercept logistic regression (null/empty model)

^b^No application of foreign material, initiation of breast feeding within 1 h of birth, feeding colostrum, no pre-lacteal feed, kept baby warm and delayed bath

One fourth of mothers practised clean cord care. Ghee was the commonest material applied (33%), followed by ash (13%) and talcum powder (12%). Application of gentian violet on the cord was 4.6% and was higher among those who had at least one ANC check-up than those who did not receive any ANC check-up and also higher among those who gave birth at home (6%) than those who delivered at a health facility (1.7%). Mothers who were earning members of the family, SHG members and those who had at least one ANC check-up were less likely to follow clean cord care when compared to non-earning mothers, non-SHG members and those who did not have even one ANC check-up. However, giving birth at a hospital had a positive association with clean cord care (Table 5). Only 35% of mothers covered the newborn immediately after birth and delayed the first bath of the baby by 48 h. Place of birth was strongly and positively associated with good thermal care, and access to communication showed some positive association with thermal care. Receiving ANC and delivering at a facility increased the likelihood of following appropriate breast-feeding practices. Further, appropriate breast-feeding practices were less likely followed by women who were least marginalized than the most marginalized women; however, this association was not statistically significant (Table 5). The effects of marginalization were similar across SHG and non-SHG households (not shown in table). Women with SHG membership or with SHG members within the household were independently associated with newborn care practices, but the direction of association for each of the outcome was inconsistent. The density of SHG households within a GP also did not have any effect on the newborn care practices (not shown in table). There was no significant association between number of contacts with community health workers and newborn care.

**Table 5:** Determinants of newborn care

Factors	Aseptic thread used for cord (only home deliveries)	Clean cord	Appropriate breast-feeding practices	Adequate thermal care
	*n* = 701 aOR^a^ (%CI) *P* value	*n* = 2208 aOR^a^ (%CI) *P* value	*n* = 2208 aOR^a^ (%CI) *P* value	(*n* = 2208) aOR^a^ (%CI) *P* value
Age of the mother in years	1.05 (0.99, 1.1)	1.02 (0.99, 1.04)	1.004 (0.98, 1.03)	1.01 (0.99, 1.03)
	0.082	0.18	0.75	0.252
Marginalization^b^				
Least	1.46 (0.76, 2.81)	0.99 (0.72, 1.36)	0.84 (0.63, 1.12)	1.04 (0.77, 1.41)
Some	1.65 (0.96, 2.79)	0.95 (0.71, 1.28)	1.07 (0.82, 1.41)	1.11 (0.84, 1.47)
Most	1	1	1	1
	0.17	0.094	0.089	0.70
Nuclear family				
Yes	1.4 (0.81, 2.4)	0.83 (0.64, 1.07)	1.04 (0.85, 1.31)	0.86 (0.68, 1.1)
No	0.23	0.16	0.73	0.23
Household size	1.03 (0.94, 1.13)	1.007 (0.97, 1.05)	0.98 (0.94, 1.01)	1.01 (0.97, 1.04)
	0.47	0.7	0.32	0.59
Woman is earning				
Yes	1.06 (0.61, 1.83)	0.56 (0.42, 0.75)	1.06 (0.83, 1.36)	0.89 (0.69, 1.15)
No	1	1	1	1
	0.83	<0.001	0.63	0.4
Access to media				
Yes	0.64 (0.39, 1.05)	0.87 (0.71, 1.11)	1.134 (0.93, 1.39)	1.23 (0.99, 1.51)
No	1	1	1	1
	0.075	0.29	0.22	0.057
SHG member				
Yes	0.97 (0.50, 1.85)	0.72 (0.56, 0.93)	1.37 (1.06, 1.76)	0.81 (0.63, 1.03)
No	1	1	1	1
	0.91	0.011	0.015	0.091
Contacts with CHW during ANC				
4 and above	1.28 (0.62, 2.64)	1.05 (0.71, 1.53)	1.49 (1.01,2.19)	1.28 (0.87, 1.86)
1–3	0.708 (0.33, 1.47)	1.26 (0.84, 1.91)	1.27 (0.84, 1.94)	1.03 (0.68, 1.56)
None	1	1	1	1
	0.07	0.30	0.08	0.16
ANC visits				
3 and above	2.95 (1.55, 5.62)	0.65 (0.47, 0.88)	1.28 (0.95, 1.74)	1.24 (0.90, 1.71)
1 or 2	2.02 (1.12, 3.7)	0.58 (0.42, 0.81)	1.58 (1.16, 2.16)	1.14 (0.84, 1.56)
None	1	1	1	1
	0.004	0.003	0.011	0.40
Place of childbirth				
Facility	–	2.6 (1.98, 3.32)	3.50 (2.74, 4.46)	4.85 (3.74, 6.27)
Home		1	1	1
		<0.001	<0.001	<0.001

^a^aOR obtained from random intercept logistic regression taking clustering at the block and GP levels

^b^Marginalization defined as a composite of caste (SC/ST = 1, others = 0), literacy (cannot read and write = 1, only read/both = 0), socio-economic status 5 (0/2 = 0, 3/4 = 1). Total score ranges from 0 to 3. 3 = most marginalized, 2 = some marginalization, 1 and 0 least marginalized. (SES score computed using PCA from household facilities and certain assets)

Mother’s awareness (at the time of interview) of newborn care was assessed. Of the 2208 mothers, around three fourths of the mothers were aware of giving birth in a health facility as a safe practice and 67% mentioned that they would take the newborn to a health facility after 1 week of birth. Only 58% were aware about initiating breast milk within 1 h of birth. Also, 9% were aware of clean cord care practice, and only 3% knew about Kangaroo mother care (KMC).

## Discussion

Our results show that institutional delivery had a significant impact on newborn care practices in the context of rural UP. Number of ANC visits was associated with breast-feeding practices in a positive way but not with clean cord care practices. The presence of an SHG member in the household, density of SHG within GP and contact with CHWs during ANC did not seem to be associated with newborn care in this population.

The focus of maternal and child health programmes have been in delivering ANC and promoting institutional delivery with the aim of reducing maternal and neonatal deaths. A population survey from Bihar showed significant relationship between newborn care and neonatal mortality [3] with odds of neonatal death increasing 3.5 times when delayed bathing practices was not followed and 2.5 times with failure to practise Kangaroo mother care. Our cross-sectional study across the rural villages of five districts of Uttar Pradesh has shown that only 5% of mothers reported to have practised all six newborn care practices correctly (clean cord care, delayed bathing (>48 h), thermal care, timely initiation of breast feeding (within 1 h), not discarding colostrum and not feeding pre-lacteal feeds). There was also variation of newborn care practices between communities, and it should be taken into consideration while implementing any intervention to optimize benefits.

In our study, antenatal care was found to be independently associated with correct breast-feeding practices and thermal care but not with clean cord care. A study from Nigeria showed that cord care education given to mothers at antenatal clinics during ANC was not associated with actual cord care and the information provided also was not standardized [16]. This same study data showed that clean cord care practice was associated with mothers’ education, gender of child (favouring males) and teaching hospital [17]. Further studies are required in states with a high burden of neonatal mortality and morbidity to assess the association of quality of ANC with cord care practices. The positive association of facility-based childbirth and newborn care practices that we demonstrate here is concordant to another study done in rural UP [8].

While there is a definitive evidence of high risk of still birth and neonatal deaths with poor socio-economic status, we did not find any association of newborn care practices with level of marginalization. This could be because newborn care is more influenced by tradition and culture and community practices rather than the socio-economic status, especially in situations like ours where nearly one third of the deliveries took place at home. This underscores the importance of the spread of appropriate messages across all strata of society rather than focusing only on the poorest.

Thermal care did not seem to have an association with the number of ANC visits irrespective of the place of childbirth. This indicates that more than the number of ANCs, it is the quality of ANCs which matter. It is also suggested that four antenatal visits may not impact the outcome of pregnancy till the coverage is 60% [2]. In our survey, only 20% of women had four or more ANC visits, which is far less than what is required.

Community health workers play a major role in imparting knowledge related to MNCH care during antenatal and postnatal period that can impact health outcomes [18, 19], and there is a compelling evidence from developing nations about the effectiveness of community health workers in improving maternal and child health [20] and specifically in improving newborn care practices. However, in our analysis, the number of community health worker visits to households during pregnancy did not have any effect on the newborn care practices. This could be due to the quality of the information exchange that happens during contact sessions. ASHA and other community link workers receive incentives for antenatal visits and delivery, but there are no incentives for delivering health promotion at the homes of these mothers. Our survey showed that around 40% of mothers were not visited by CHWs in the postnatal period. There is a need for improving the quality of training, supportive supervision and adequate follow-up. Given that the time spent by mothers after childbirth in facilities is not more than 24 h and the inability to devote quality time by hospital staff due to excess workload, this gap should be compensated by ensuring regular home visit by a community health worker to sensitize mothers regarding newborn care especially during the immediate postnatal period. The presence of CHWs is a huge opportunity existing in communities. Efforts are underway to train them in order to promote newborn care practices. However, there is a lack of monitoring and accountability [10]. The lack of an apparent impact could be because of the fact that existing CHWs were not fully trained when the data were collected.

One of the key limitations of this study is the length of recall period (15 months) of newborn care practices and antenatal care during pregnancy. The recalled practices could have been influenced by the other information the mother would have gained before the survey. Hence, the percentage of respondents reporting good newborn care practices could be an overestimate as shown by the discrepancy between the awareness of clean cord care (9%) and reported clean cord care practice (25%). We did not collect information at the GP or block level which could have allowed us to explore reasons for cluster-level factors affecting newborn care practices. Despite its limitations, this data provides insights into the actual practices on a large sample of population in one of the states with poor health indicators. Locally collected data would help frame an intervention package that would have greater acceptability by the population.

In this sample of population, an inconsistent association was found between SHG membership and newborn care practices. At the time of the survey, SHGs were not utilized for improving awareness about healthy mother and child practices and this could explain the lack of this association. However, there is evidence that self-help groups can have an impact on improving health care utilization and practices [21, 22]. In our setting, the SHG platform could be a potential resource to improve community behaviours and may provide a more sustainable solution to a problem that the state is grappling with.

## Conclusion

Based on the survey findings, we can conclude that there is a need for improving newborn care among the rural population living in Uttar Pradesh. This would impact on reduction of neonatal morbidity and mortality. While the thrust by the existing government programmes for the promotion of at least four antenatal care visits and institutional deliveries should continue, it is time that the quality of care offered in the facilities and the counselling given by community health workers both during antenatal and the immediate postnatal period be stepped up. Mobilizing self-help groups and strengthening women’s linkages with community health workers may be promising, and the evaluation of this community mobilization project could provide some evidence in the context of rural UP.

## Abbreviations

ANC: Antenatal care
CHWs: Community health workers
GP: Gram panchayat
RGMVP: Rajiv Gandhi Mahila Vikas Pariyojana
SES: Socio-economic status
SHGs: Self-help groups
UP: Uttar Pradesh
